# Construction of a Novel MYC-Associated ceRNA Regulatory Network to Identify Prognostic Biomarkers in Colon Adenocarcinoma

**DOI:** 10.1155/2022/3216285

**Published:** 2022-07-05

**Authors:** Rui Xin, Xiao-Mei Tang, Ying-Jie Jiang, Fei Yu, Sha Li, Cheng-You Jia, Gao-Ren Wang, Da Fu, Ji-Bin Liu, Yu-Shui Ma

**Affiliations:** ^1^Department of Nuclear Medicine, Shanghai Tenth People's Hospital, Tongji University School of Medicine, Shanghai 200072, China; ^2^Institute of Oncology, Affiliated Tumor Hospital of Nantong University, Nantong 226631, Jiangsu, China; ^3^Department of General Surgery, Institute of Pancreatic Diseases, Ruijin Hospital, Shanghai Jiaotong University School of Medicine, Shanghai 200025, China; ^4^Department of Pathology, Navy Military Medical University Affiliated Changhai Hospital, Shanghai 200433, China; ^5^Cancer Institute, Longhua Hospital, Shanghai University of Traditional Chinese Medicine, Shanghai 200032, China

## Abstract

Colorectal cancer (CRC) includes colon adenocarcinoma (COAD) and rectal adenocarcinoma (READ). Competitive endogenous RNA (ceRNA) is crucial for cancer pathogenesis. Abnormal expression of MYC is generally associated with a poor colon adenocarcinoma prognosis. The present study aimed to identify a novel MYC-associated ceRNA regulatory network and identify potential prognostic markers associated with COAD. We obtained the transcriptome sequencing profiles of 462 COAD cases from the TCGA database and analyzed differentially expressed genes (DEGs) in MYC high expression (MYC^high^) and MYC low expression (Myc^low^) tumors. We identified an important lncRNA, LINC00114, which effectively predicts overall survival and plays a protective role in COAD. Moreover, the LINC00114/miR-216a-5p axis was identified as a clinical prognostic model. The predicted target genes of the LINC00114/miR-216a-5p axis include uromodulin Like 1 (UMODL1) and oncoprotein induced transcript 3 (OIT3), which are closely related to the survival and prognosis of COAD patients. In summary, we constructed a novel ceRNA regulatory network and identified potential biomarkers for the targeted therapy and prognosis of COAD.

## 1. Introduction

Colorectal cancer (CRC) is a widely occurring cancer worldwide with an increasing rate of incidence [1, 2]. The cause of CRC is complex and involves several genetic and environmental factors [3]. CRC is a concern for the global population due to its high recurrence rate [4]. To date, even combination therapy has been unable to improve the prognosis of CRC patients [5, 6]. Hence, understanding the biology behind the manifestation of CRC is necessary to design effective therapeutic strategies against it.

Colon adenocarcinoma (COAD) is a type of CRC [7] and globally ranks 3rd and 4th in the incidence and mortality rate of cancer, respectively [8, 9]. Although progress has been made in its early detection and treatment, the overall survival rate of advanced COAD is not satisfactory [10–12].

The competitive endogenous RNA (ceRNA) network hypothesis reveals a novel mechanism of RNA interaction [13]. Several noncoding RNAs (ncRNAs), including microRNA (miRNA) and long noncoding RNA (lncRNA), may be associated with cancer and can be part of the ceRNA network [14, 15]. Previous studies have shown that the ceRNA network, including ncRNAs like hsa-circ-000984 and miR-145, participates in the metastasis and prognosis of CRC [16].

The MYC family of genes is a group of early-discovered oncogenes regarded as promising anticancer targets [17–19]. Abnormal expression of MYC is generally associated with a poor cancer prognosis. The dysregulation in gene expression is not only due to chromosomal translocations or copy number alterations involving the MYC gene, but also because MYC is located downstream of multiple oncogene signaling pathways. For example, the dysregulated WNT signaling pathway in colorectal tumors always results in high MYC levels. Thus, MYC expression above the physiologically allowed threshold can induce tumor development or strongly accelerate tumorigenesis in multiple tissues [20].

The Cancer Genome Atlas (TCGA) uses innovative genome analysis techniques for a comprehensive understanding of cancer genetics and helps produce novel cancer therapies, diagnostic techniques, and prevention strategies [21]. Among the prognostic biomarkers related to COAD, most are not experimentally or prospectively validated [22, 23]. This warrants more research for elucidating potential biomarkers for the detection and treatment of COAD.

**Figure 1:**
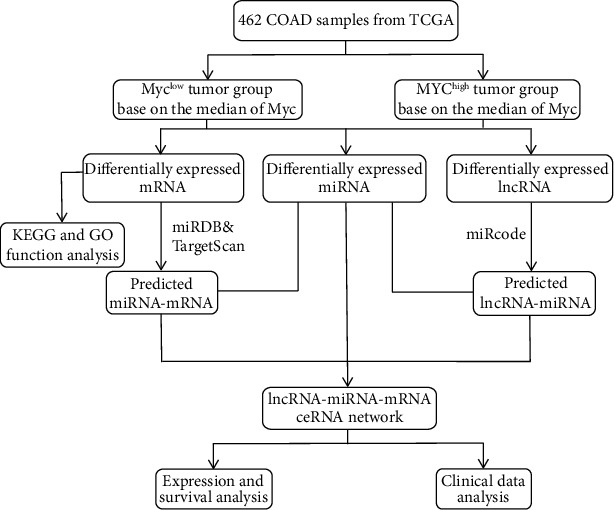
Flow chart of MYC-related ceRNA network construction in COAD.

In this study, we want to construct a novel ceRNA network related to MYC in COAD. Firstly, through differential expression analysis in two groups of MYC^high^ and Myc^low^ expression (based on the median value of MYC expression) in 462 COAD samples, the lncRNA-miRNA-mRNA triple regulatory networks constructed from three differentially expressed RNAs were obtained. A functional enrichment analysis was conducted to assess the functional role and potential mechanism of the network in COAD. Then, a key ceRNA network was identified by expression analysis, survival analysis, and nuclear-cytoplasmic localization analysis of RNAs from hub-triple regulatory networks. Finally, Cox regression analysis was carried out to obtain the diagnostic and prognostic value of UMODL1/OIT3 for COAD, GO, and KEGG analysis were utilized to obtain the possible function of UMODL1/OIT3 in COAD. Methylation analysis and immune infiltration analysis were further performed to study the potential biological function of UMODL1/OIT3 in COAD. Hence, we believe that our study will be helpful in understanding the probable underlying molecular mechanism and help in the clinical prediction and treatment of COAD (Figure 1).

## 2. Methods

### 2.1. Data Processing and Analysis

A total of 462 lncRNAs/mRNAs and 438 miRNAs sequencing data of COAD patients were obtained from the TCGA website (https://portal.gdc.cancer.gov). All raw RNA-Seq data (lncRNA, miRNA, and mRNA) was normalized as fragments per kilobase of exon model per million mapped fragment reads. Transformation of miRNA sequences into human mature miRNA names using the Starbase v2.0 database (https://starbase.sysu.edu.cn) [24]. We used the Cancer Cell Line Encyclopedia (CCLE, https://portals.broadinstitute.org/ccle) to verify the expression level of cancer cell lines and the Human Protein Atlas (HPA) (https://www.proteinatlas.org) for verification of the protein level of ceRNAs. We obtained gene mutation status with cBioPortal (https://www.cbioportal.org). The tumor samples were divided into two groups, namely, MYC^high^ (*n* = 231) and Myc^low^ (*n* = 231), according to the median expression level of MYC. We identified the differentially expressed lncRNAs, miRNAs, and mRNAs with the thresholds of |logFC| > 0.5 and *P* < 0.05. Volcano maps were visualized with the GraphPad Prism 8 software (version 8.4.2). Heatmaps were drawn with TBtools software (version 0.655).

### 2.2. Construction and Identification of the ceRNA Network

LncRNAs play important roles in cells, such as binding to chromatin or mRNA, or to miRNAs or proteins (“sponge” effect) [25]. We constructed the ceRNA network by the following steps: (1) the miRcode database (https://www.mircode.org) was used to explore all DEmiRNAs that interact with DElncRNA [26]. (2) Using the miRDB (https://www.mirdb.org/) and TargetScan (https://www.targetscan.org) databases, we predicted the target mRNAs of DEmiRNAs [27]. (3) The R software was utilized to compare the target genes with DEmRNAs, and the target genes that overlapped with DEmRNAs in this study were selected for the next analysis. (4) We determined the location of DElncRNAs in cells using the LncLocator database (https://www.csbio.sjtu.edu.cn/bioinf/lncLoc-ator) [28]. (5) CeRNA networks were visualized using the “Cytoscape” software (https://www.cytoscape.org) [29]. (6) We visualized the “pathways” through bubble graphs and presented the KEGG analysis results using the R software package “ggplot2.” Furthermore, the “Cytoscape” plug-in “cytoHubba” was applied to find the hub-triple regulatory network.

### 2.3. Methylation and Expression Analysis

Studies have shown that DNA methylation is a significant epigenetic mechanism that is able to regulate gene expression and influence the behavior of cancer cells [30]. UALCAN (https://ualcan.path.uab.edu) was used to analyse the degree of methylation of target genes. MethSurv: a web tool to perform multivariable survival analysis using DNA methylation data (https://biit.cs.ut.ee/methsurv) was used to obtain the CpG methylation data of target genes. MEXPRESS (https://mexpress.be) was used for visualizing TCGA and methylation expression and clinical information.

### 2.4. Immune Infiltration Level and Expression Analysis

To investigate the association between the expression of target genes and tumor-infiltrating immune cells, we applied TIMER2.0 (https://timer.cistrome.org), which is an online tool for the analysis and visualization of the correlation between immune infiltrate levels and a number of variables across diverse cancer types. We explored the correlation of target gene expression with the abundance of tumor-infiltrating immune cells, the prognostic value, and target gene copy numbers in COAD. Furthermore, we estimated the correlation of target genes with the typical markers of 16 tumor-infiltrating immune cells. The top 20 genes (PCC/Pearson's *r* > 0.4) associated with target genes were obtained from GEPIA (https://gepia.cancer-pku.cn/) are shown in Figures S4D and S5D.

### 2.5. Statistical Analysis

The obtained data were analyzed using the SPSS 23.0 software (SPSS Inc, Chicago, IL, USA). The data were visualized using the GraphPad Prism (version 8.0). The results of the correlation and survival analyses of the lncRNA-miRNA-mRNA network were expressed as the median and 95% CI. The Mann–Whitney test and independent *t*-test were used to calculate differences between the two groups of data, while one-way ANOVA with the Kruska–Wallis test and the chi square test were utilized to evaluate the difference among different groups. A *P*value <0.05 was considered statistically significant.

## 3. Results

### 3.1. The Role of MYC Overexpression in COAD

**Figure 2:**
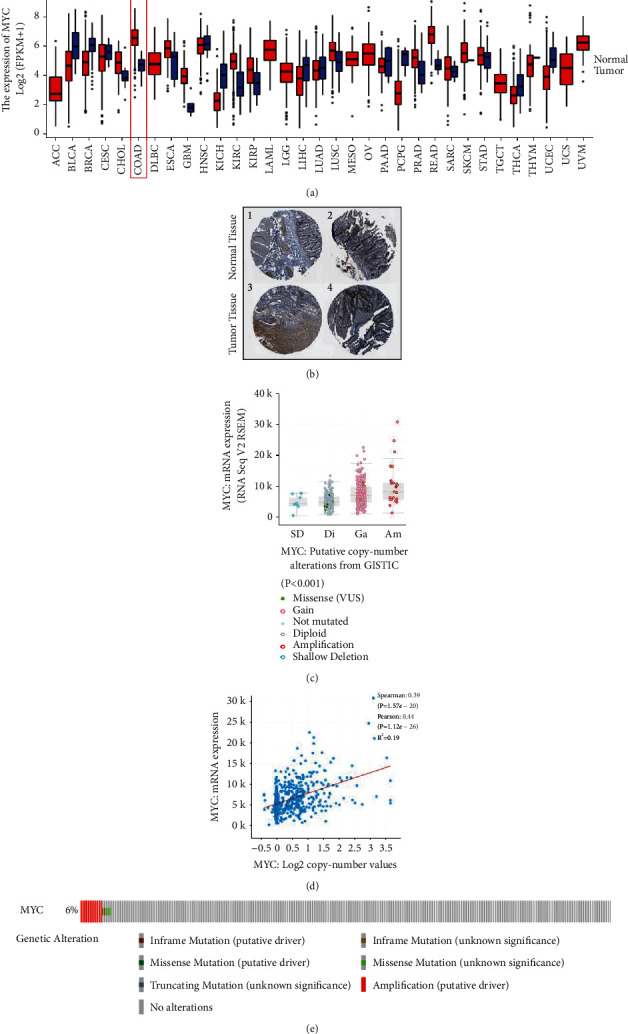
The functional characteristics of MYC in COAD. (a) Expression and distribution of MYC in pan-cancer tissues. (b) Verifying the expression of MYC on translational level through The Human Protein Atlas database (immunohistochemistry). (c) MYC copy number and mRNA expression. (d) The correlation between MYC copy number and mRNA expression. (e) The distribution of MYC genome changes in the TCGA data set.

According to the TCGA and HPA database, MYC expression was higher in COAD tissues than in normal tissues (*P* < 0.001) (Figures 2(a) and S1A). Immunohistochemical (IHC) staining obtained from the HPA database confirmed a similar level of MYC expression (Figure 2(b)). IHC analysis of the patient data is shown in Supplementary Table 1. The MYC expression distribution in pan-cancer cell lines and the clinical tumor–node–metastasis (TNM) stage of MYC were shown in the CCLE (Figures S1B and S1C). Furthermore, genetically altered regions of MYC in COAD were mainly expressed through amplification (*P* < 0.001) (Figure 2(c)). A positive correlation was found between the MYC copy value and mRNA expression in COAD samples (*P* < 0.001) (Figure 2(d)). The distribution of MYC genomic changes in COAD is shown in Figure 2(e).

### 3.2. Identifying DEGs in COAD

**Figure 3:**
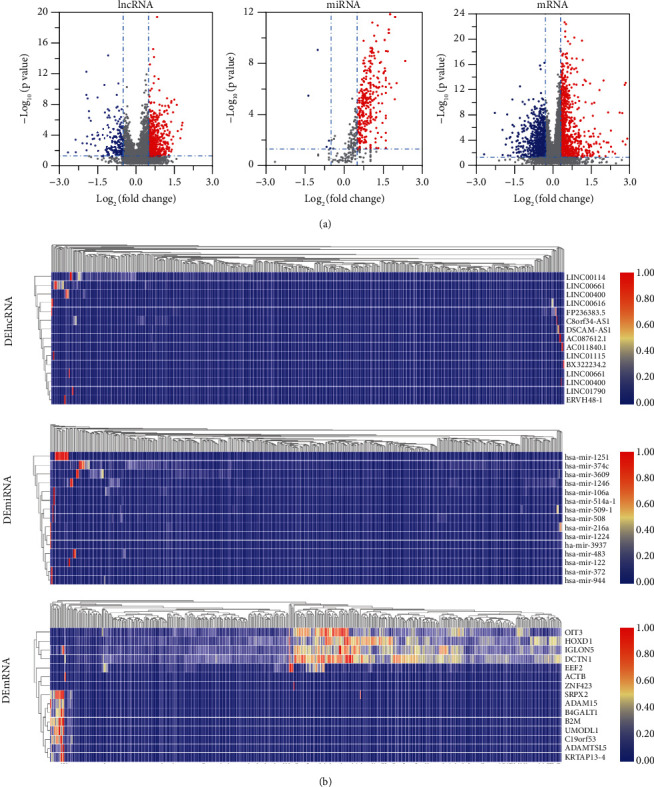
Identification of differential genes. (a) Volcano maps of differently expressed ln cRNAs, miRNAs, and mRNAs between two groups: MYChigh group versus MYClow group in CRC; (b) the heatmap of differentially expressed lncRNAs (up), miRNAs (middle), an d mRNAs (down). Red and blue spots represented significant upregulated and downregulated RNAs, respectively; DElncRNAs: differentially expressed long noncoding RNAs; DEmiRNA: differentially expressed microRNA; DEmRNA: differentially expressed messenger RNA.

Based on the abovementioned results, we speculated that a MYC-related ceRNA network could serve as a potential prognostic model for COAD. To verify this hypothesis, we downloaded all the data of COAD patients from the TCGA database and divided into two groups (MYC^high^ and Myc^low^) based on the median MYC expression level. Subsequently, a total of 907 DElncRNAs (653 upregulated and 254 downregulated), 337 DEmiRNAs (331 upregulated and 6 downregulated), and 9240 DEmRNAs (7311 upregulated and 1929 downregulated) were screened from the COAD samples. We constructed volcano plots and heatmaps to show the distribution of DERNAs and describe the 15 significant DERNAs, respectively (Figure 3).

### 3.3. Functional Enrichment Analysis of DEmRNAs

**Figure 4:**
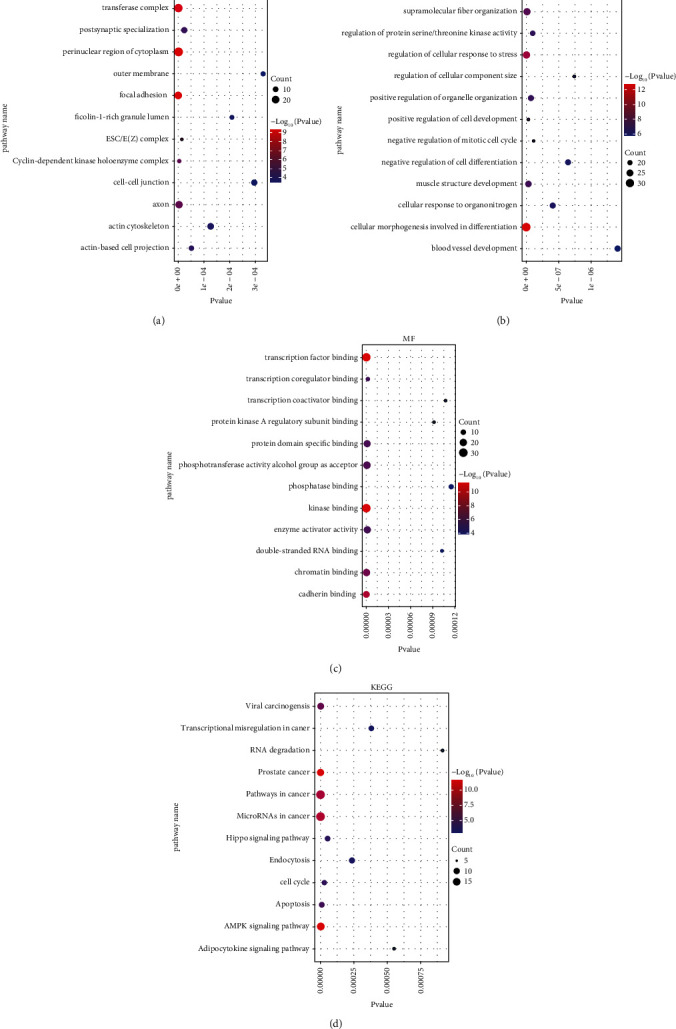
Functional enrichment analysis of the DE mRNAs in the network. (a) CC of DE mRNAs. (b) BP of DE mRNAs. (c) MF of DE mRNAs. (d) KEGG pathway of DE mRNAs.

We used the Metascape database to investigate the functions of all DEmRNAs from Gene Ontology (GO) and KEGG pathway analysis (Figure 4). The most enriched GO terms were “transferase complex,” “supermolecule fiber organization,” and “transcription factor binding,” respectively. The KEGG pathway enrichment analysis showed that the “pathways in cancer,” “transcriptional misregulation in cancer,” and “microRNAs in cancer” were most involved in COAD.

### 3.4. Construction of the MYC-Associated Triple Regulatory Network

**Figure 5:**
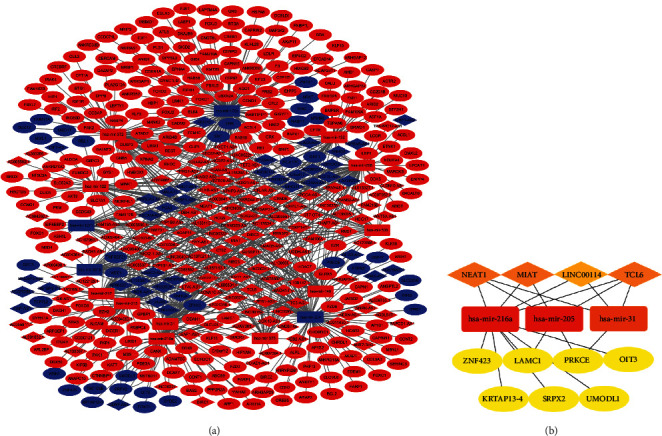
Construction of lncRNA-miRNA-mRNA triple regulatory networks in COAD. (a) Triple regulatory networks in COAD, where “red“ means up and “blue” means down. (b) The 14 hub ceRNAs.

To establish a MYC-associated triple regulatory network of lncRNA-miRNA-mRNA in COAD, we conducted a comprehensive analysis of the high- and low-MYC expression groups. First, put all DElncRNAs into the “miRcode” database to identify potential miRNAs targeting lncRNAs. However, only 15 out of the predicted miRNAs were selected after taking the intersection with DEmiRNAs. Subsequently, we used the TargetScan database to identify downstream target mRNAs through these 15 DEmiRNAs. The “Cytoscape” was used to visualize the lncRNA-miRNA-mRNA triple regulatory network (Figure 5(a)). Finally, the “Cytoscape” plug-in “cytoHubba” was utilized to determine the hub genes. The results show that four lncRNA (NEAT1, MIAT, LINC00114, and TCL6), three miRNAs (hsa-mir-216a, hsa-mir-205, and hsa-mir-31), and seven mRNAs (ZNF423, LAMC1, PRKCE, OIT3, KRTAP13-4, SRPX2, and UMODL1) were identified as part of the regulatory network (Figure 5(b)).

### 3.5. Construction and Verification of ceRNA Network

**Figure 6:**
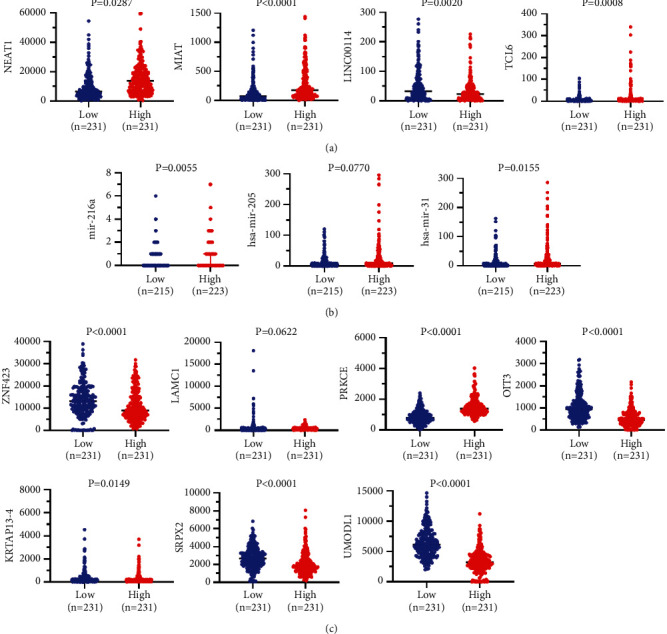
Expression of 14 hub ceRNAs. (a) Four DElncRNAs. (b) Three DEmiRNAs. (c) Seven hub DEmRNAs.

**Figure 7:**
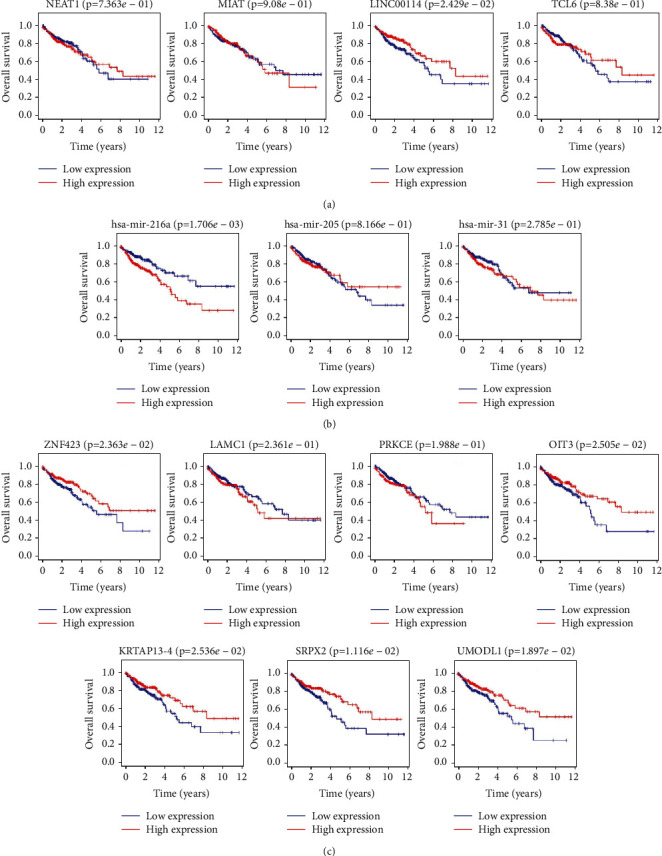
Overall survival analysis of the hub ceRNAs. (a) Four DElncRNAs. (b) Three DEmiRNAs. (c) Seven hub DEmRNAs.

**Figure 8:**
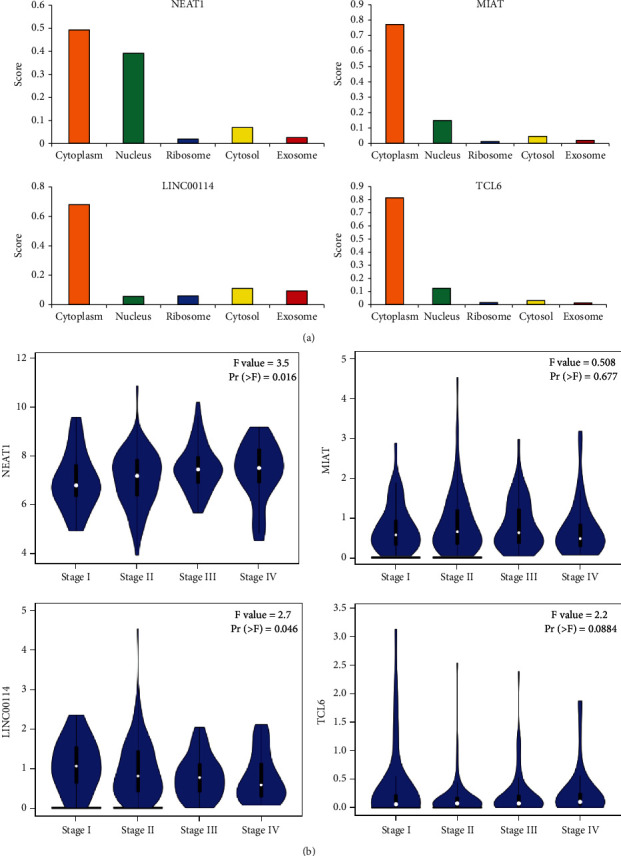
The cellular localization (a) and clinical pathological stages (b) for lncRNAs.

**Figure 9:**
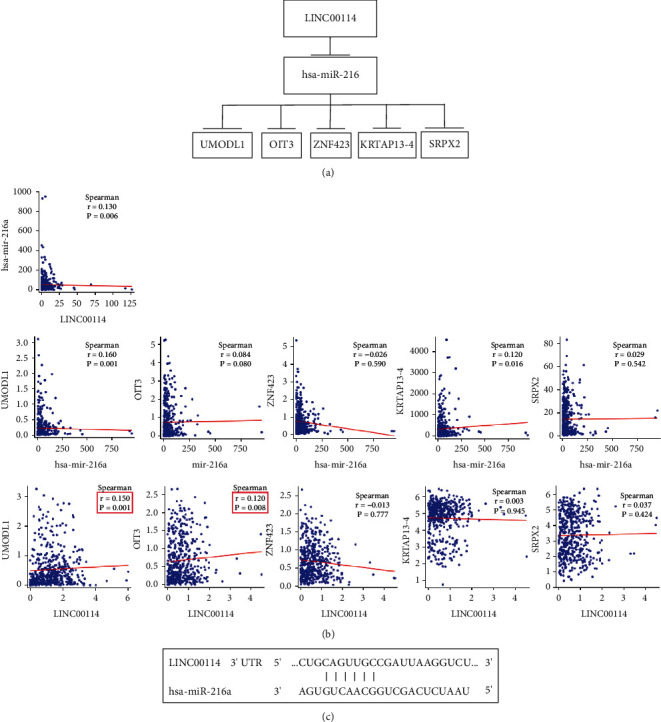
Construction and verification of the ceRNA network. (a) The predicted triple regulatory network in COAD. (b) Correlation analysis between predictive ceRNAs. (c) Predictive base pairing between the target sites of miRNA and lncRNA in the 3' UTR.

We assessed the expression levels of DERNAs from the hub of triple regulatory networks in COAD samples. We observed four downregulated lncRNAs (NEAT1, MIAT, LINC00114, and TCL6), three upregulated miRNAs (hsa-mir-216a, hsa-mir-205, and hsa-mir-31), and seven downregulated mRNAs (ZNF423, LAMC1, PRKCE, OIT3, KRTAP13-4, SRPX2, and UMODL1) in COAD samples with MYC^high^ and Myc^low^ expression groups (Figure 6). Then, to determine whether these RNAs were associated with COAD prognosis, we used Kaplan-Meier analysis and a log-rank test to perform an overall survival (OS) analysis of COAD patients. The OS analysis of COAD patients showed one DElncRNA (LINC00114), one DEmiRNA (hsa-mir-216a), and five DEmRNAs (ZNF423, OIT3, KRTAP13-4, SRPX2, and UMODL1) related to the prognosis of COAD (Figure 7). Furthermore, the four hub lncRNAs were mainly located in the cytoplasm (Figure 8(a)). We next explored the clinicopathological stages of the four hub lncRNAs. With the deterioration of tumor invasion, the expression of LINC00114 decreased statistically (Figure 8(b)). Therefore, the lncRNA-miRNA-mRNA network included one lncRNA, one miRNA, and five mRNAs (Figure 9(a)). Through the expression correlation analysis, a positive correlation between LINC00114 and UMODL1/OIT3 expression was observed (Figure 9(b)). As shown in Figure S2, we analyzed the correlation between predictive ceRNAs and MYC in COAD. Positive correlations between MYC and LINC00114/UMODL1/OIT3 expressions were observed. The 3′ UTR binding locations of LINC00114 and hsa-miR-216a are shown in Figure 9(c). These data indicated that LINC00114, as a ceRNA, may regulate mRNA expression by regulating hsa-miR-216a.

### 3.6. Clinical Relevance of LINC00114-UMODL1/OIT3 Axis in COAD Patients

To understand the relationship between the expression level of LINC00114/UMODL1/OIT3 and clinical features, we performed a correlation analysis (Supplementary Tables 2–4). The expression level of LINC00114 positively correlated with the TNM stage, diameter of the tumor, lymph node metastasis, distant metastasis, and body mass index (BMI) (Supplementary Table 5). OIT3 expression levels were significantly correlated with BMI (Supplementary Table 7). However, no significant correlation between the expression level of UMODL1 and clinical factors was found (Supplementary Table 6). In addition, the OS characteristics of COAD patients in the TCGA cohort were found by univariate and multivariate Cox regression analysis. LINC00114 (HR = 0.650, *P*=0.033) expression was significantly associated with poor prognosis (Table S8). The TNM stage, tumor diameter, and distant metastasis were relevant to the OS of COAD patients (Tables S9 and S10). Furthermore, through the multivariate Cox regression analysis, we proved that LINC00114 expression was still relevant to OS in COAD patients (HR = 0.640, *P*=0.028) (Table S8). In summary, LINC00114 may become an independent prognostic factor for COAD patients.

### 3.7. Expression of UMODL1 and OIT3 in Various Cancers

The GEPIA database was used to evaluate UMODL1 and OIT3 expression in human cancer. Figures S3A and S3B show the UMODL1 and OIT3 expression profiles across all tumor samples and paired normal tissues. UMODL1 expression was low in most cancers, except in acute myeloid leukemia and thymoma (Figure S3A). Furthermore, the expression of OIT3 was significantly low in liver hepatocellular carcinoma (*P* < 0.001) (Figure S3B). Depending on the CCLE, UMODL1 and OIT3 were low in various cancer cell lines, including COAD cell lines (Figures S3C and S3D). The distribution of the genomic changes in UMODL1/OIT3 is shown on the cBioPortal OncoPrint plot (Figures S4A and S5A). However, no significant association was found between UMODL1/OIT3 expression and the copy number value among COAD samples (Figures S4B and S5B). Most of the COAD samples harbored a diploid UMODL1/OIT3 (Figures S4C and S5C). Consistently, COAD samples harboring an OIT3 deletion exhibited lower mRNA expression than those with diploid OIT3 (*P* < 0.001). We used the GO and KEGG pathway enrichment analysis of the most correlated genes of UMODL1 and OIT3 in COAD (Figures S4D and S5D). The KEGG enrichment term most relevant to UMODL1 was “Signaling pathways regulating pluripotency of stem cells,” while the UMODL1-related GO enrichment analysis was mainly enriched in “positive regulation of apoptotic signaling pathway,” “cell projection membrane,” and “p53 binding” (Figure S6). Moreover, enrichment terms related to OIT3 were enriched in “positive regulation of the canonical Wnt signaling pathway,” “the intracellular protein-containing complex,” and “DNA-binding transcription factor binding” (Figure S7).

### 3.8. Relationship between Methylation and Expression of UMODL1/OIT3

**Figure 10:**
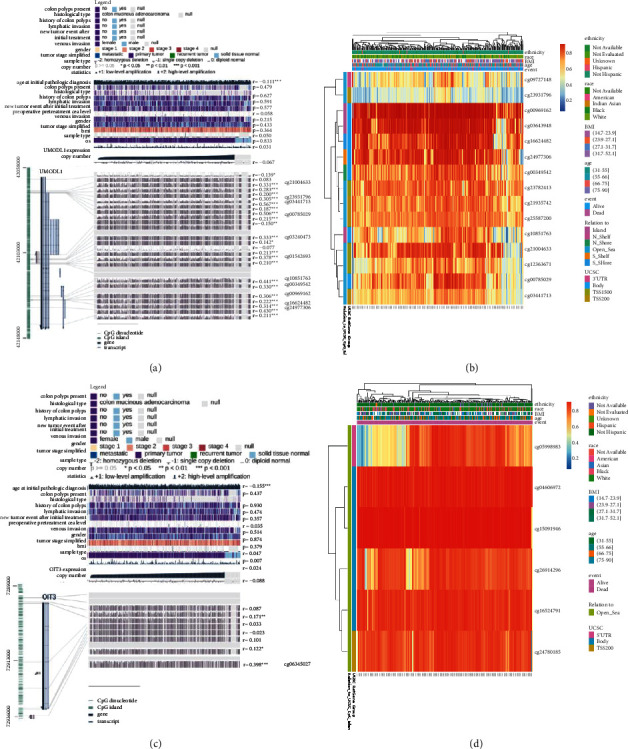
Methylation analysis of UMODL1 and OIT3. (a) The methylation sites of UMODL1 DNA sequences association with gene expression. (b) Different methylated regions associated with UMODL1. (c) The methylation sites of OIT3 DNA sequences association with gene expression. (d) Different methylate d regions associated with OIT3.

The methylation level of UMODL1/OIT3 is shown in Figure S8. According to the MEXPRESS database, remarkable methylation of UMODL1/OIT3 in the clinical factor of “age at initial pathologic diagnosis” was observed (Figures 10(a) and 10(c)). Methylation of UMODL1 occurred at multiple sites, including cg21004633, cg23931796, cg03441713, cg00785029, cg03240473, cg01542693, cg10851763, cg00349542, cg00969162, cg16624482, and cg24977306 (*r* = 0.331, 0.305, 0.567, 0.506, 0.333, 0.378, 0.441, 0.330, 0.306, 0.314, and 0.430, respectively) (Figure 10(a)). Methylation of OIT3 occurred at cg06345027 (*r* = 0.398) (Figure 10(c)). We also used MethSurv to identify the differential methylation regions related to UMODL1/OIT3 and clinical factors of patients with a heatmap. Most of the UMODL1/OIT3-associated methylation sites were in the gene body region and TSS200 region (Figures 10(b) and 10(d)).

### 3.9. Correlation between the Expression of Predicted Target Genes and Immune Infiltration

**Figure 11:**
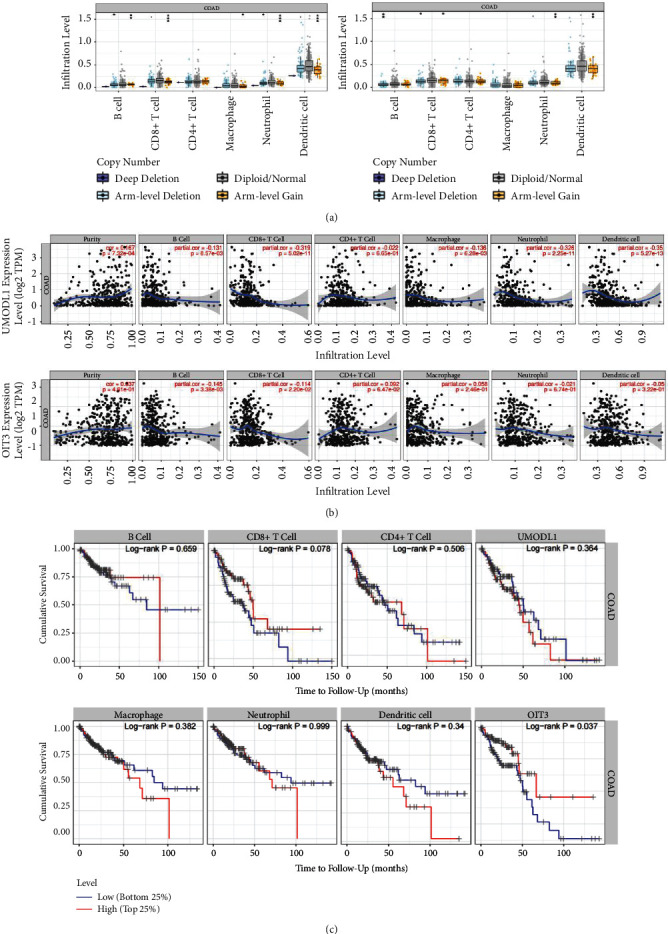
Correlation analysis between the expression of UMOLD1/OIT3 and immune infiltration in COAD. (a) Gene copy number and immune cell infiltration levels. (b) Gene expression and immune infiltration level. (c) Immune infiltration and overall survival.

The characteristics of tumor-infiltrating immune cells are closely connected with the occurrence of cancer [31, 32]. Using the “SCNA” module, analysis demonstrated that the infiltration levels of B cells, CD8+ T cells, neutrophils, macrophages, and dendritic cells in COAD were likely related to the change of UMODL1 gene copy number (Figure 11(a)), while the infiltration levels of B cells, CD8+ T cells, neutrophils, and dendritic cells were correlated with the copy number of OIT3 (Figure 11(a)). “Gene” module analysis showed that UMODL1 expression significantly correlated with tumor purity, B cells, CD8+ T cells, macrophages, neutrophils, and dendritic cells in COAD (*P* < 0.05) (Figure 11(b)). Furthermore, OIT3 expression was closely related to the infiltration level of B cells and CD8+ T cells in COAD (*P* < 0.05) (Figure 11(b)). Several markers of natural killer cells, dendritic cells, Th1 cells, and T cell exhaustion were significantly and positively correlated with UMODL1 expression in COAD (*P* < 0.05) (Table S11). Markers of STAT5B (Treg) and GZMB (T cell exhaustion) had positive correlations with OIT3 expression in COAD (*P* < 0.05) (Table S11). When the relationship between UMODL1/OIT3 expression and the markers in the GEPIA database were verified, the results showed a similar trend (*P* < 0.001) (Tables S12 and S13). In conclusion, tumor-infiltrating immune cells may affect the clinical consequences of LINC00114/UMODL1 and LINC00114/OIT3 axes in COAD.

## 4. Discussion

CRC can be cured at earlier stages, but the prognosis of advanced CRC is poor. Hence, the early prevention or detection of CRC is critical [33]. READ and COAD are two different types of CRC based on the anatomical location [34–36]. Identifying potential biomarkers and therapeutic targets of COAD is crucial for improving the prognosis of this disease. Recently, the ceRNA hypothesis increased our understanding of oncogenesis [37, 38].

There is a growing number of pathophysiological roles for the MYC family in various cancers, including COAD [39–41]. We established a MYC-related ceRNA triple network in colon adenocarcinoma from the experience of our previous ceRNA network construction for liver cancer [42]. Firstly, by comparing the MYC^high^ tumor tissues with Myc^low^ tumor tissues, we identified 907 DElncRNAs, 337 DEmiRNAs, and 9204 DEmRNAs. Through the hub analysis of “Cytoscape,” a triple key regulatory network was obtained, including four lncRNAs, three miRNAs, and seven mRNAs. Following that, we evaluated the expression and survival of the hub regulatory networks by performing a subcellular localization analysis of the four lncRNAs. Meanwhile, we also analyzed 14 DERNAs in the ceRNA network through Cox regression, methylation, and immune infiltration analysis. Finally, we obtained a LINC00114-miR-216a-UMODL1/OIT3 axis associated with the prognosis of COAD.

LncRNAs are relevant to the development of COAD, and the lncRNA LINC00114 is a potential target for the diagnosis of COAD [43]. Another study identified three COAD-related lncRNAs with prognostic values (LINC00114, LINC00261, and HOTAIR) [44]. In addition, LINC00114 may be associated with the OS of CRC patients [45]. LINC00114 inhibited CRC progression via miRNA miR-133b sponging [46]. Similarly, through univariate and multivariate Cox regression analyses, we proved the relevance of LINC00114 expression in the OS of COAD patients (Table S8). We also demonstrated that LINC00114 might be an independent prognostic factor for COAD patients.

COAD patients with a high expression of mir-216a-5p often show poor OS [47], consistent with the results of our study. The expression of mir-216a-5p is significantly downregulated in COAD and correlates with each stage of tumor differentiation [48]. Furthermore, miR-216a-3p inhibits COX-2 and ALOX5 expression in COAD cells, thereby affecting the proliferation of COAD cells [49]. However, contrary to the above results, some studies have shown that miR-216a acts as a tumor suppressor. The miRNA is expressed by the TGF-*β*/MAP1S pathway and can inhibit autophagy [50]. In gastric cancer, miR-216a is significantly upregulated [51]. In conclusion, the precise role of miR-216a in tumorigenesis needs to be further studied.

Aberrant methylation has long been considered a hallmark of cancer. Therefore, we used several databases to explore possible explanations for the abnormal expression of predicted target genes at DNA methylation levels in COAD. According to the MEXPRESS database, the methylation of UMODL1/OIT3 was associated with the clinical factor of “age at initial pathologic diagnosis.” In addition, we found abnormal UMODL1/OIT3 DNA methylation in COAD, with more hypermethylation sites closer to open sea regions according to MethSurv. All OIT3-related methylation sites were located in the open sea region. Therefore, we can conclude that abnormal methylation of UMODL1/OIT3 may be relevant to the poor prognosis of COAD.

The characteristics of tumor-infiltrating immune cells are related to the occurrence of cancer [52]. The present study showed that several immune cell infiltration levels are negatively associated with the copy number of the UMODL1/OIT3 gene in COAD. The expression of UMODL1/OIT3 was highly associated with the immune infiltration of COAD. Many types of tumor-infiltrating immune cells are significantly related to the prognosis of COAD patients. In addition, UMODL1/OIT3 expression showed a significant positive correlation with some immune marker groups derived from dendritic cells. T helper cells. These findings collectively indicate that the differences induced by UMODL1/OIT3 may affect the tumor immune microenvironment and the development of COAD. However, upon further evaluation, we did not find a significant correlation between immune infiltration and the OS of COAD.

To better understand the biological functions of UMODL1 and OIT3, GO, and KEGG enrichment analyses were conducted. The most relevant KEGG enrichment term of UMODL1 was “Signal pathways regulating pluripotency of stem cells.” GO enrichment analysis related to UMODL1 was mainly enriched in “positive regulation of apoptotic signaling pathway,” “cell projection membrane,” and “p53 binding.” Recent studies have confirmed the expression of UMODL1 in the immune system. After being stimulated by the CD3/CD28 antibody, UMODL1 shows a fast response in proliferating CD4+ T cells, indicating that it impacts the immune defense against pathogens [53].

A comprehensive analysis of gene expression found novel genes related to CRC, including OIT3, which may be a new marker for this cancer [54]. In CRC, mutations can affect important pathways and genes, such as c-MYC, PIK3CA, and PTEN, which can be used to predict the prognosis of CRC patients [55, 56].

However, this study has some limitations. The lack of articles and experimental evidence suggests that our knowledge of LINC00114/UMODL1/OIT3 is far from complete, and their properties and functions remain largely unknown [57]. The data for lncRNA, miRNA, and mRNA obtained from the database should be validated through *in vitro* and *in vivo* experiments. Furthermore, more research is needed to discover effective biomarkers and targets for the diagnosis and treatment of CRC.

In conclusion, we analyzed COAD sequencing data from TCGA to reveal key ceRNAs associated with MYC and evaluated their diagnostic and prognostic potential to find novel and reliable biomarkers for COAD. We constructed a novel MYC-associated ceRNA regulatory network of COAD and identified potential biomarkers for precisely targeted therapy and prognosis. We found a critical lncRNA (LINC00114), which effectively predicts the prognosis and survival of COAD patients and plays a protective role in CRC. Meanwhile, the LINC00114/miR-216a-5p axis was identified as a clinical prognostic model and their target genes, including UMODL1 and OIT3, are closely related to the survival and prognosis of COAD patients. We believe that our findings will help understand the potential molecular mechanism and provide new insights for the clinical prediction and treatment of COAD. Furthermore, key RNAs significantly related to the prognosis of COAD can be developed as potential prognostic and diagnostic biomarkers for COAD.

## Data Availability

The datasets analyzed during the current study are available from the corresponding author on reasonable request.

## Supplementary Materials

Supplementary Materials Supplementary Figure S1 : functional characteristics of MYC in COAD. Supplementary Figure S2 : correlation analysis between 7 predictive ceRNAs and MYC in COAD. Supplementary Figure S3 : UMODL1 and OIT3 expressed levels in pan-cancer. Supplementary Figures S4 and S5 : genomic alterations of UMODL1/OIT3 in COAD. Supplementary Figures S6 and S7 : functional enrichment analysis of UMODL1/OIT3 and related genes in COAD. Supplementary Figure S8 : evaluation of methylation. (A) UMODL1. (B) OIT3. Supplementary Table S1 : IHC of MYC in Human Protein Atlas database. Supplementary Table S2–S4 : relationship between LINC00114/UMODL1/OIT3 expression and clinicopathologic parameters of COAD patients. Supplementary Table S5–S7 : the association between the expression levels of LINC00114/UMODL1/OIT3 and clinical factors. Supplementary Table S8–S10 : univariate and multivariate analyses of clinicopathological characteristics in COAD patients. Supplementary Table S11 : correlation analysis between UMODL1 and biomarkers of immune cells in COAD. Supplementary Tables S12 and S13 : correlation analysis between UMODL1/OIT3 and biomarkers in CRC (Supplementary Materials).
