# Supplementary material for: Construction of a Novel MYC-Associated ceRNA Regulatory Network to Identify Prognostic Biomarkers in Colon Adenocarcinoma
Source: J Oncol. 2022 Jul 5;2022:3216285. doi: 10.1155/2022/3216285 (PMC9277212; doi:10.1155/2022/3216285)
Supplement: Supplementary Materials — Supplementary Figure S1 : functional characteristics of MYC in COAD. Supplementary Figure S2 : correlation analysis between 7 predictive ceRNAs and MYC in COAD. Supplementary Figure S3 : UMODL1 and OIT3 expressed levels in pan-cancer. Supplementary Figures S4 and S5 : genomic alterations of UMODL1/OIT3 in COAD. Supplementary Figures S6 and S7 : functional enrichment analysis of UMODL1/OIT3 and related genes in COAD. Supplementary Figure S8 : evaluation of methylation. (A) UMODL1. (B) OIT3. Supplementary Table S1 : IHC of MYC in Human Protein Atlas database. Supplementary Table S2–S4 : relationship between LINC00114/UMODL1/OIT3 expression and clinicopathologic parameters of COAD patients. Supplementary Table S5–S7 : the association between the expression levels of LINC00114/UMODL1/OIT3 and clinical factors. Supplementary Table S8–S10 : univariate and multivariate analyses of clinicopathological characteristics in COAD patients. Supplementary Table S11 : correlation analysis between UMODL1 and biomarkers of immune cells in COAD. Supplementary Tables S12 and S13 : correlation analysis between UMODL1/OIT3 and biomarkers in CRC (Supplementary Materials). [file 3216285.f1.docx]

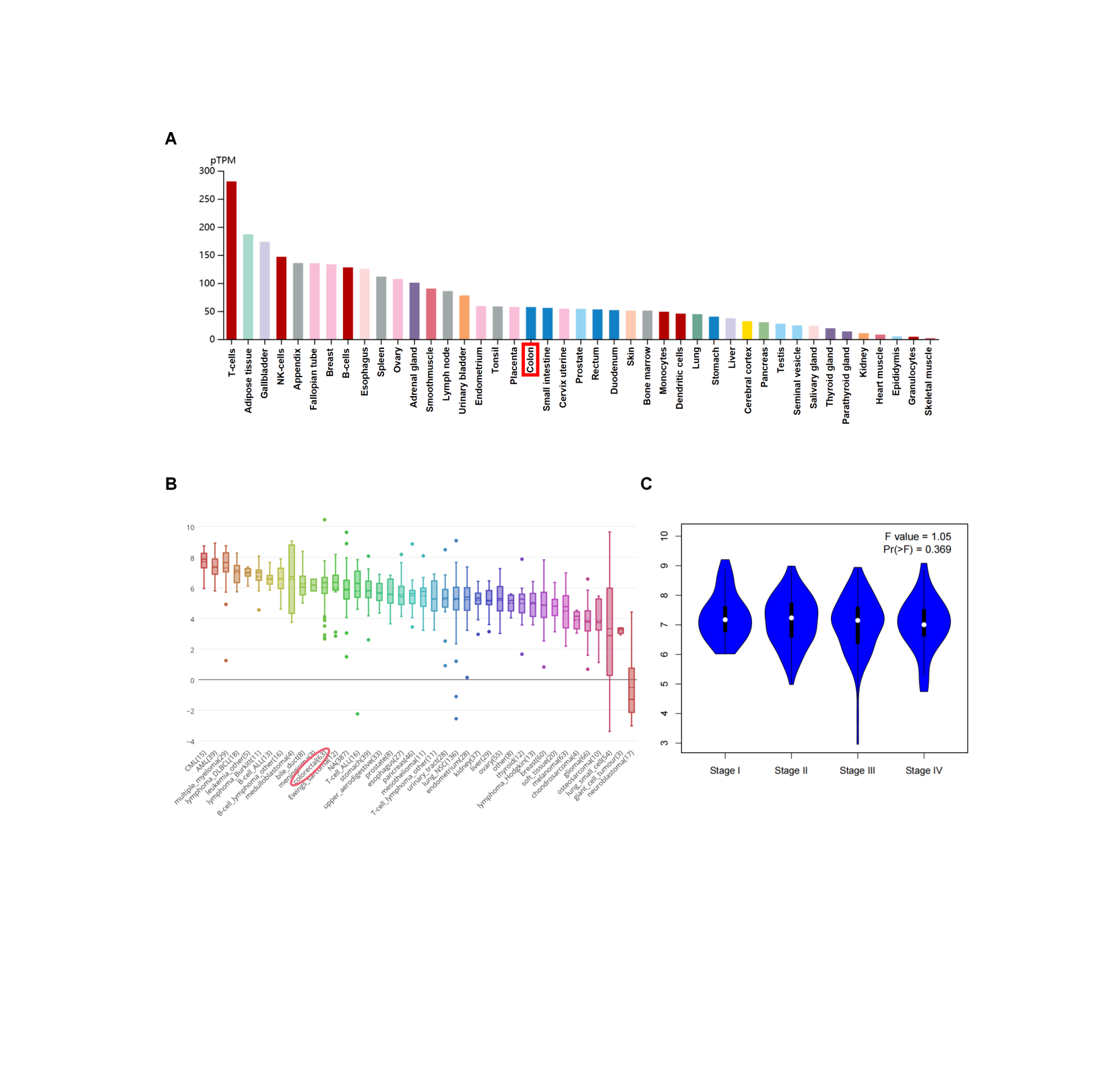


**Figure S1. Functional characteristics of MYC in COAD.** (A) The expression distribution of MYC in pan-cancer tissues by the HPA database. (B) The expression distribution of MYC in pan-cancer cell lines in CCLE. (C) The expression distribution of MYC between TNM stages.


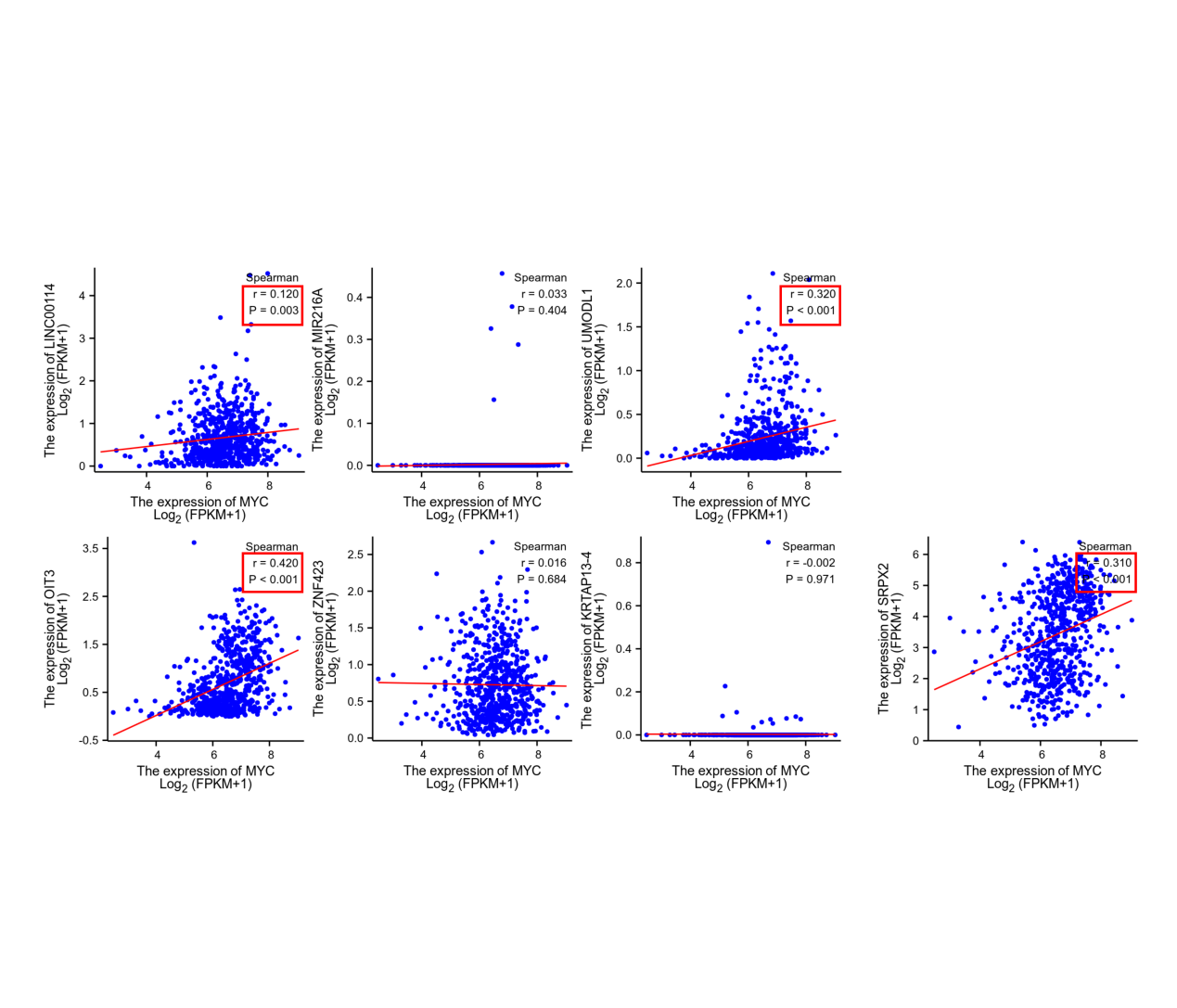


**Figure S2. Correlation analysis between 7 predictive ceRNAs and MYC in COAD.**
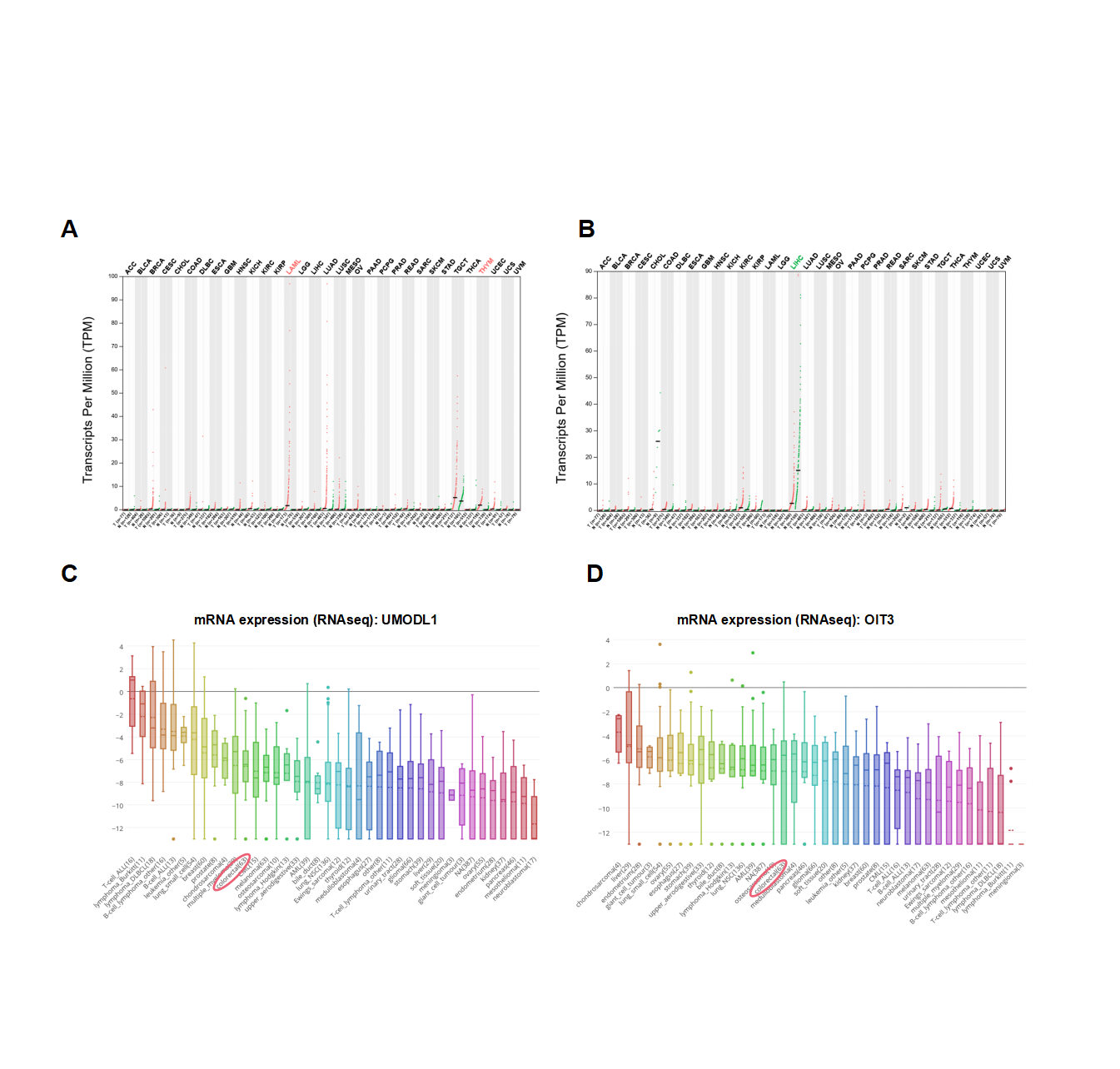


**Figure S3. The UMODL1 and OIT3 expressed levels in pan-cancer.** (A,B) The gene expression profile across all tumor samples and paired normal tissues. (C,D) the expression distribution of UMODL1 and OIT3 in pan-cancer cell lines.


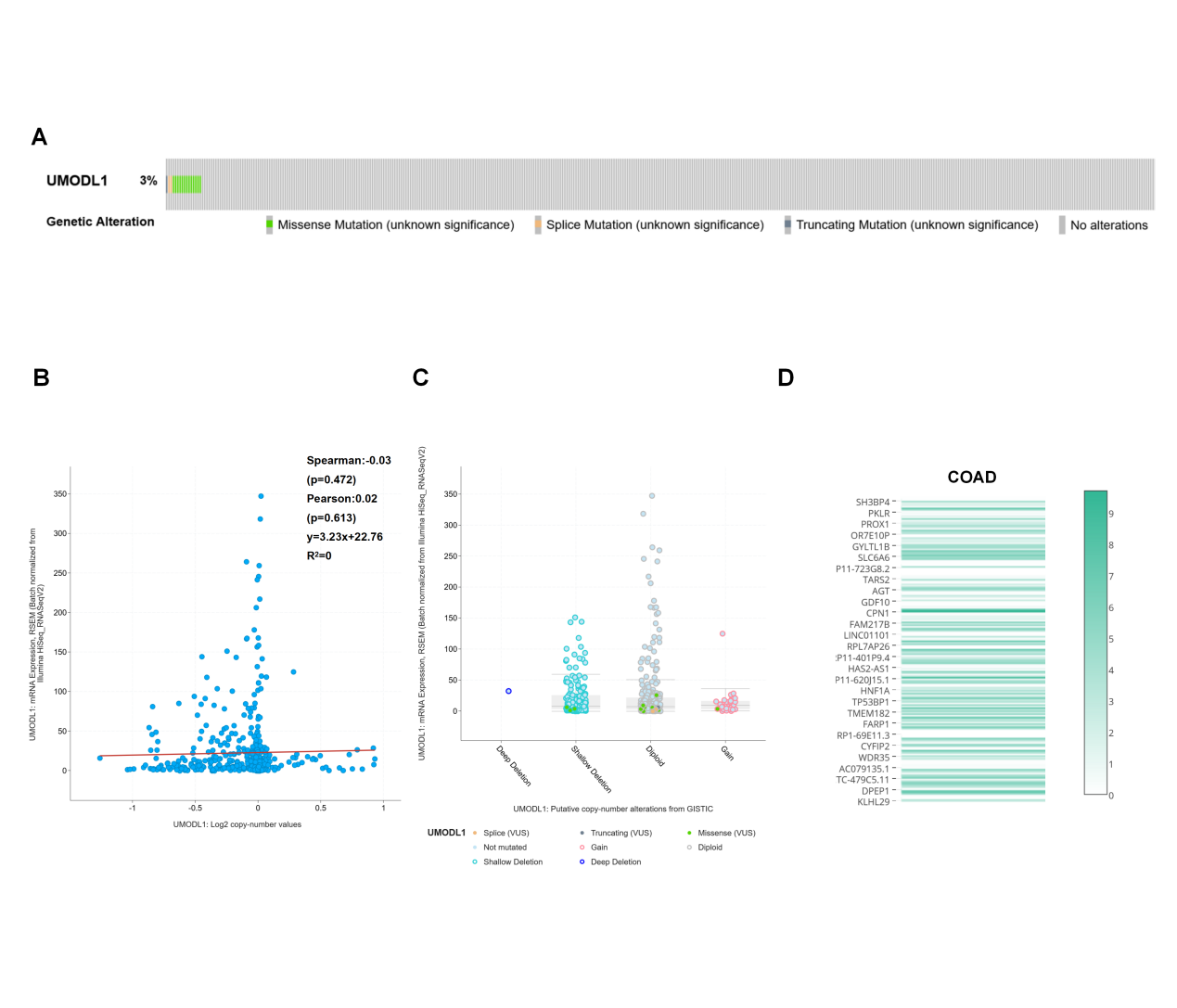


**Figure S4. Genomic alterations of UMODL1 in COAD.** (A) The distribution of UMODL1 genome changes in the TCGA dataset. (B) The correlation between UMODL1 copy number and mRNA expression. (C) UMODL1 copy number and mRNA expression. (D) The correlated genes of UMODL1 in COAD.


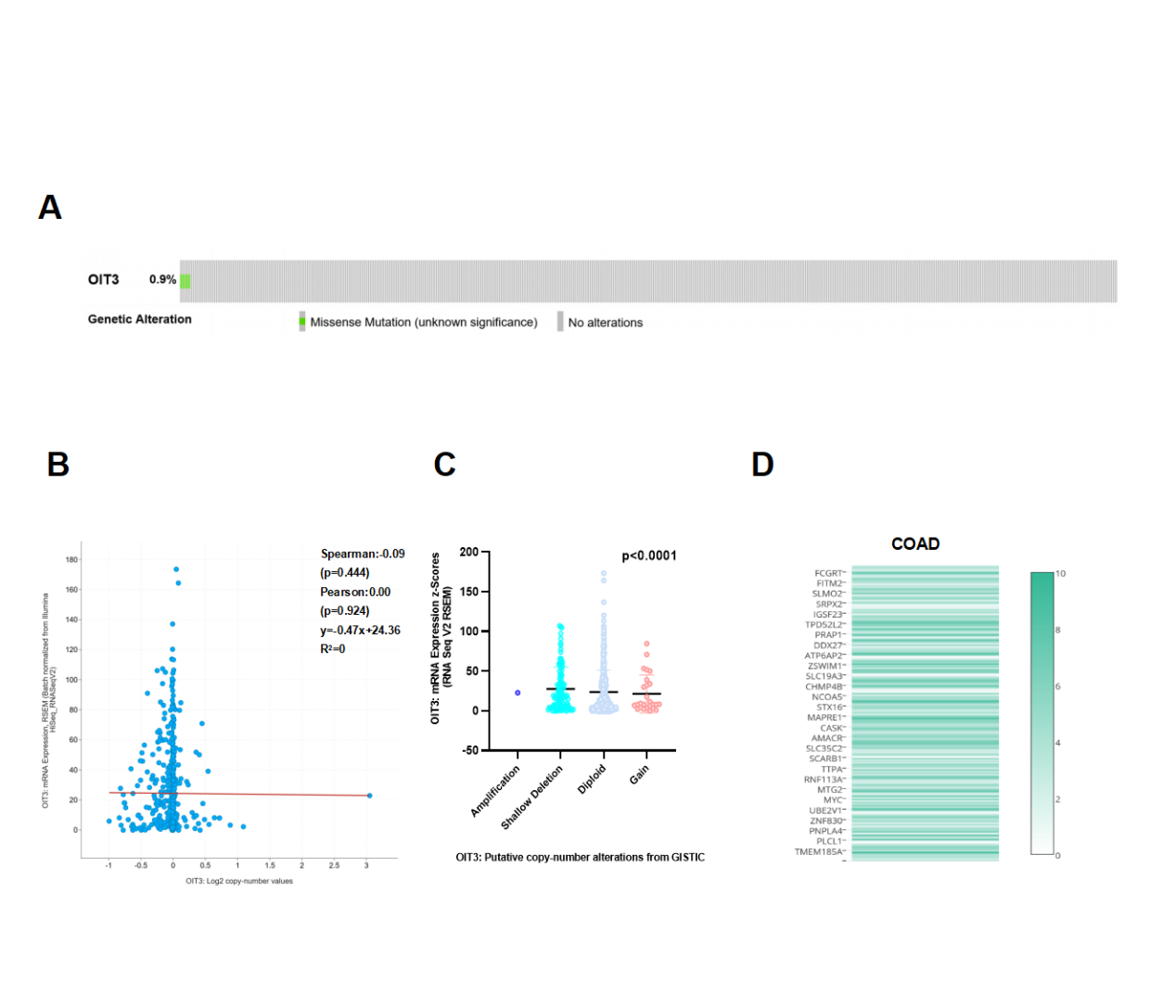


**Figure S5. Genomic alterations of OIT3 in COAD.** (A) The distribution of OIT3 genome changes in the TCGA dataset. (B) The correlation between OIT3 copy number and mRNA expression. (C) OIT3 copy number and mRNA expression. (D) The correlated genes of OIT3 in COAD.


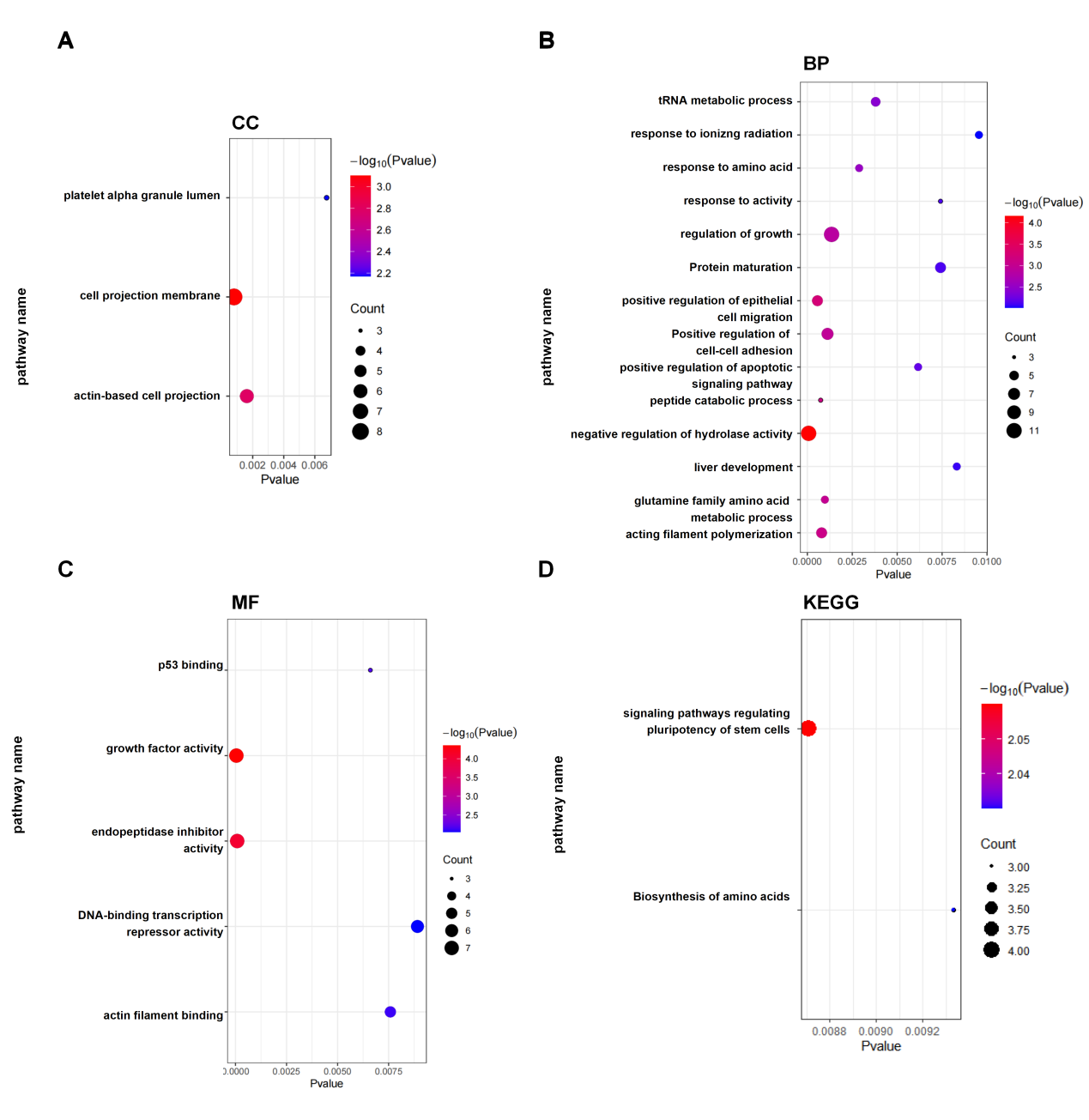


**Figure S6. Functional enrichment analysis of UMODL1 and related genes in COAD.** (A) CC of DE mRNAs. (B) BP of DE mRNAs. (C) MF of DE mRNAs. (D) KEGG pathway of DE mRNAs.


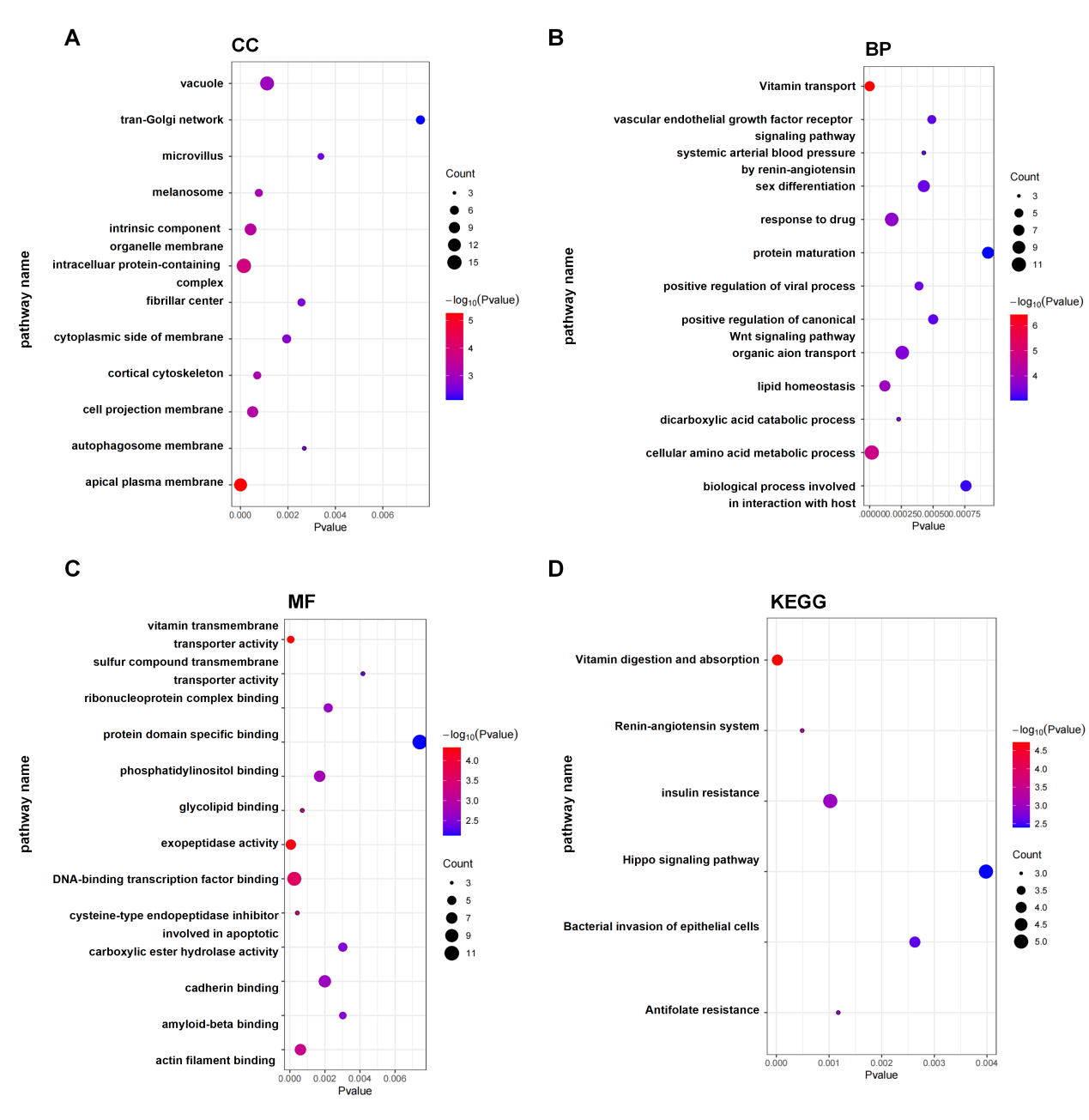


**Figure S7. Functional enrichment analysis of OIT3 and related genes in COAD.** (A) CC of DE mRNAs. (B) BP of DE mRNAs. (C) MF of DE mRNAs. (D) KEGG pathway of DE mRNAs.


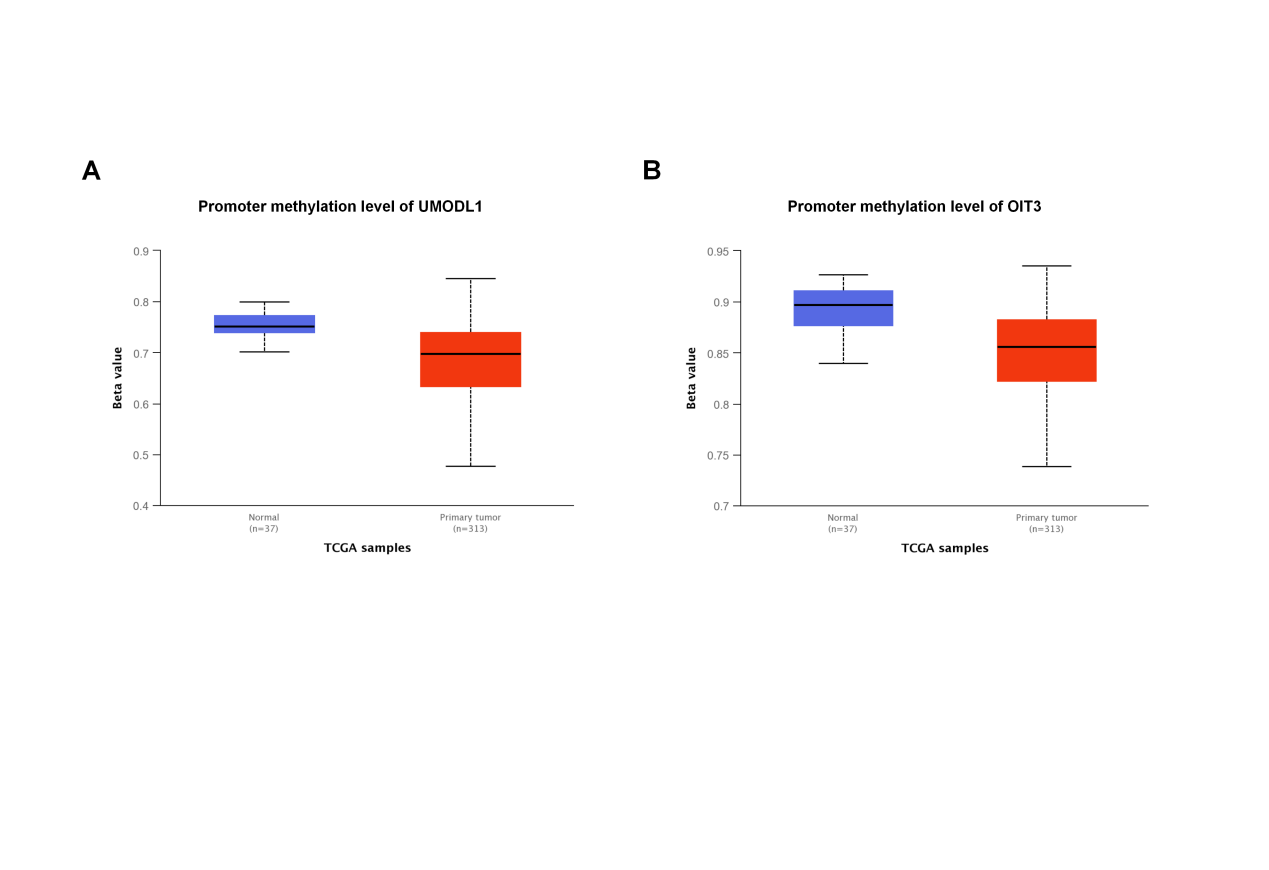


**Figure S8. Evaluation of methylation.** (A) UMODL1. (B) OIT3.

**Table S1. IHC of MYC in Human Protein Atlas database.**

| **No.** | **Tissue type** | **ID** | **Age** | **Gender** | **staining** |
| --- | --- | --- | --- | --- | --- |
| **1** | Colorectal cancer | 2944 | 80 | Female | Low |
| **2** | Colorectal cancer | 3266 | 73 | Male | Low |
| **3** | Colorectal cancer | 1958 | 84 | Female | Medium |
| **4** | Colorectal cancer | 2096 | 72 | Male | Medium |

**Table S2. Relationship between LINC00114 expression and clinicopathologic parameters of COAD patients.**

| **Characteristics** | | **Number of cases** | **LINC00114 expression** | | | **P value** |
| --- | --- | --- | --- | --- | --- | --- |
|  |  |  | **Low** | **high** | |  |
| **Age** | <60 | 135 | 56 | | 79 | 0.059 |
|  | ≥60 | 316 | 169 | | 147 |  |
| **Gender** | Male | 239 | 114 | | 125 | 0.323 |
|  | Female | 212 | 111 | | 101 |  |
| **TNM Stage** | I+II | 251 | 114 | | 137 | **0.003** |
|  | III+IV | 189 | 109 | | 80 |  |
|  | Unknown | 11 | 2 | | 9 |  |
| **Diameter** | T1+T2 | 89 | 37 | | 52 | **0.079** |
|  | T3+T4 | 362 | 188 | | 174 |  |
| **Lymph-node  metastasis** | 266 | 119 | 147 | | 266 | **0.009** |
|  | 185 | 106 | 79 | | 185 |  |
| **Distant  metastasis** | Negative | 331 | 162 | | 169 | **0.001** |
|  | Positive | 63 | 44 | | 19 |  |
|  | Unknown | 57 | 19 | | 38 |  |
| **BMI** | ≤18.4 | 1 | 1 | | 0 | **0.009** |
|  | 18.5-23.9 | 61 | 30 | | 31 |  |
|  | 24-27.9 | 64 | 22 | | 42 |  |
|  | ≥28 | 106 | 47 | | 59 |  |
|  | unkown | 219 | 125 | | 94 |  |

|  |
| --- |

|  |
| --- |

**Table S3. Relationship between UMODL1 expression and clinicopathologic parameters of COAD patients.**

| **Characteristics** | | **Number of cases** | **UMODL1 expression** | | | **P value** |
| --- | --- | --- | --- | --- | --- | --- |
|  |  |  | **Low** | **high** | |  |
| **Age** | <60 | 135 | 66 | | 69 | 0.781 |
|  | ≥60 | 316 | 159 | | 157 |  |
| **Gender** | Male | 239 | 112 | | 127 | 0.172 |
|  | Female | 212 | 113 | | 99 |  |
| **TNM Stage** | I+II | 251 | 123 | | 128 | 0.570 |
|  | III+IV | 189 | 97 | | 92 |  |
|  | Unknown | 11 | 8 | | 3 |  |
| **Diameter** | T1+T2 | 89 | 42 | | 47 | 0.201 |
|  | T3+T4 | 362 | 183 | | 179 |  |
| **Lymph-node  metastasis** | 266 | 266 | 130 | | 136 | 0.604 |
|  | 185 | 185 | 95 | | 90 |  |
| **Distant  metastasis** | Negative | 331 | 163 | | 168 | 0.884 |
|  | Positive | 63 | 32 | | 31 |  |
|  | Unknown | 57 | 30 | | 27 |  |
| **BMI** | ≤18.4 | 1 | 1 | | 0 | 0.532 |
|  | 18.5-23.9 | 61 | 29 | | 32 |  |
|  | 24-27.9 | 64 | 30 | | 34 |  |
|  | ≥28 | 106 | 49 | | 57 |  |
|  | unkown | 219 | 116 | | 103 |  |

|  |
| --- |

|  |
| --- |

**Table S4. Relationship between OIT3 expression and clinicopathologic parameters of COAD patients.**

| **Characteristics** | | **Number of cases** | **OIT3 expression** | | | **P value** |
| --- | --- | --- | --- | --- | --- | --- |
|  |  |  | **Low** | **high** | |  |
| **Age** | <60 | 135 | 69 | | 66 | 0.734 |
|  | ≥60 | 316 | 156 | | 160 |  |
| **Gender** | Male | 239 | 115 | | 124 | 0.424 |
|  | Female | 302 | 110 | | 192 |  |
| **TNM Stage** | I+II | 251 | 118 | | 133 | 0.159 |
|  | III+IV | 189 | 99 | | 90 |  |
|  | Unknown | 11 | 8 | | 3 |  |
| **Diameter** | T1+T2 | 89 | 39 | | 50 | 0.201 |
|  | T3+T4 | 362 | 186 | | 176 |  |
| **Lymph-node  metastasis** | 266 | 266 | 129 | | 137 | 0.478 |
|  | 185 | 185 | 96 | | 89 |  |
| **Distant  metastasis** | Negative | 331 | 156 | | 175 | 0.149 |
|  | Positive | 63 | 36 | | 27 |  |
|  | Unknown | 57 | 33 | | 24 |  |
| **BMI** | ≤18.4 | 1 | 1 | | 0 | 0.246 |
|  | 18.5-23.9 | 61 | 36 | | 25 |  |
|  | 24-27.9 | 64 | 33 | | 31 |  |
|  | ≥28 | 106 | 56 | | 50 |  |
|  | unkown | 219 | 100 | | 110 |  |

|  |
| --- |

|  |
| --- |

**Table S5. The association between the expression levels of LINC00114 and clinical factors.**

| **Characteristics** | **Spearman** | **P value** |
| --- | --- | --- |
| **Age** | -.110* | 0.051 |
| **Gender** | -0.047 | 0.324 |
| **TNM Stage** | -0.083 | **0.078** |
| **Diameter** | -0.082 | **0.008** |
| **Lymph-node  metastasis** | **-.124^**^** | **0.009** |
| **Distant  metastasis** | -0.002 | **0.002** |
| **BMI** | **-.124^**^** | **0.009^*^** |

**Table S6. The association between the expression levels of UMODL1 and clinical factors.**

| **Characteristics** | **Spearman** | **P value** |
| --- | --- | --- |
| **Age** | -0.013 | 0.782 |
| **Gender** | -0.064 | 0.173 |
| **TNM Stage** | -0.017 | 0.715 |
| **Diameter** | -0.027 | 0.571 |
| **Lymph-node  metastasis** | -0.024 | 0.605 |
| **Distant  metastasis** | -0.023 | 0.632 |
| **BMI** | -0.051 | 0.284 |

**Table S7. The association between the expression levels of OIT3 and clinical factors.**

| **Characteristics** | **Spearman** | **P value** |
| --- | --- | --- |
| **Age** | 0.016 | 0.735 |
| **Gender** | -0.038 | 0.425 |
| **TNM Stage** | -0.072 | 0.126 |
| **Diameter** | -0.060 | 0.202 |
| **Lymph-node  metastasis** | -0.033 | 0.479 |
| **Distant  metastasis** | -0.091 | 0.053 |
| **BMI** | **-.093^*^** | **0.048^*^** |

**Table S8. Univariate and munivariate analyses of clinicopathological characteristics in COAD patients.**

| **Characteristics** |  | **Univariate Cox** |  |  | **Munivariate Cox** |  |
| --- | --- | --- | --- | --- | --- | --- |
|  | **HR** | **95% CI** | **P value** | **HR** | **95% CI** | **P value** |
| **Age** | 1.406 | 0.889-2.225 | 0.145 |  |  |  |
| **Gender** | 0.923 | 0.623-1.367 | 0.690 |  |  |  |
| **TNM stage** | 2.428 | 1.751-3.367 | **0.001*** | 2.044 | 1.425-2.933 | **0.001*** |
| **Diameter** | 2.967 | 1.374-6.406 | **0.006*** | 2.388 | 1.103-5.170 | **0.027*** |
| **Lymph-node metastasis** | 2.645 | 1.770-3.952 | **0.001*** |  |  |  |
| **Distant metastasis** | 1.583 | 1.257-1.995 | **0.001*** | 1.426 | 1.099-1.850 | **0.008*** |
| **BMI** | 1.067 | 0.892-1.277 | 0.477 |  |  |  |
| **LINC00114**  **expression** | 0.650 | 0.438-0.965 | **0.033*** | 0.640 | 0.430-0.954 | **0.028*** |

HR, Hazard ratio; CI, Confidence interval. Bold fonts indicate statistically significant values (*P<0.05). **Table S9. Univariate and munivariate analyses of clinicopathological characteristics in COAD patients.**

| **Characteristics** |  | **Univariate Cox** |  |  | **Munivariate Cox** |  |
| --- | --- | --- | --- | --- | --- | --- |
|  | **HR** | **95% CI** | **P value** | **HR** | **95% CI** | **P value** |
| **Age** | 1.406 | 0.889-2.225 | 0.145 |  |  |  |
| **Gender** | 0.923 | 0.623-1.367 | 0.690 |  |  |  |
| **TNM stage** | 2.428 | 1.751-3.367 | **0.001*** | 2.040 | 1.431-2.910 | **0.001*** |
| **Diameter** | 2.967 | 1.374-6.406 | **0.006*** | 2.490 | 1.151-5.385 | **0.008*** |
| **Lymph-node metastasis** | 2.645 | 1.770-3.952 | **0.001*** |  |  |  |
| **Distant metastasis** | 1.583 | 1.257-1.995 | **0.001*** | 1.375 | 1.064-1.777 | **0.019*** |
| **BMI** | 1.067 | 0.892-1.277 | 0.477 |  |  |  |
| **UMODL1**  **expression** | 0.872 | 0.590-1.28 | 0.492 |  |  |  |

HR, Hazard ratio; CI, Confidence interval. Bold fonts indicate statistically significant values (*P<0.05). **Table S10. Univariate and munivariate analyses of clinicopathological characteristics in COAD patients.**

| **Characteristics** |  | **Univariate Cox** |  |  | **Munivariate Cox** |  |
| --- | --- | --- | --- | --- | --- | --- |
|  | **HR** | **95% CI** | **P value** | **HR** | **95% CI** | **P value** |
| **Age** | 1.406 | 0.889-2.225 | 0.145 |  |  |  |
| **Gender** | 0.923 | 0.623-1.367 | 0.690 |  |  |  |
| **TNM stage** | 2.428 | 1.751-3.367 | **0.001*** | 2.165 | 1.521-3.082 | **0.001*** |
| **Diameter** | 2.967 | 1.374-6.406 | **0.006*** | 2.618 | 1.210-5.666 | **0.005*** |
| **Lymph-node metastasis** | 2.645 | 1.770-3.952 | **0.001*** |  |  |  |
| **Distant metastasis** | 1.583 | 1.257-1.995 | **0.001*** | 1.411 | 1.090-1.826 | **0.012*** |
| **BMI** | 1.067 | 0.892-1.277 | 0.477 |  |  |  |
| **OIT3 expression** | 1.114 | 0.753-1.647 | 0.589 |  |  |  |

HR, Hazard ratio; CI, Confidence interval. Bold fonts indicate statistically significant values (*P<0.05). **Table S11. Correlation analysis between UMODL1 and biomarkers of immune cells in COAD.**

| **Description** | **Gene markers** | **OIT3** | | | **UMODL1** | |
| --- | --- | --- | --- | --- | --- | --- |
|  |  | **Cor** | | **Pvalue** | **Cor** | **Pvalue** |
| CD8+ T cell | CD8A | -0.095349002 | | 0.04138714 | -0.095349002 | 0.04138714 |
|  | CD8B | 0.104805407 | | 0.0248978 | 0.104805407 | 0.0248978 |
| T cell (general) | CD3D | -0.032407164 | | 0.489044036 | -0.032407164 | 0.489044036 |
|  | CD3E | -0.047879255 | | 0.3065693 | -0.047879255 | 0.3065693 |
|  | CD2 | -0.054106163 | | 0.247842832 | -0.054106163 | 0.247842832 |
| B cell | CD19 | 0.044628236 | | 0.340614969 | 0.044628236 | 0.340614969 |
|  | CD79A | 0.05179774 | | 0.268625456 | 0.05179774 | 0.268625456 |
| Monocyte | CD86 | -0.029834187 | | 0.524205298 | -0.029834187 | 0.524205298 |
|  | CD115 (CSF1R) | 0.05654374 | | 0.227144012 | 0.05654374 | 0.227144012 |
| TAM | CCL2 | 0.072147109 | | 0.123120742 | 0.072147109 | 0.123120742 |
|  | CD68 | -0.048855661 | | 0.296798843 | -0.048855661 | 0.296798843 |
|  | IL10 | 0.035410094 | | 0.44966398 | 0.035410094 | 0.44966398 |
| M1 Macrophage | INOS (NOS2) | 0.00956988 | | 0.838158615 | -0.223260937 | 1.39E-06 |
|  | IRF5 | 0.137591847 | | 0.003171648 | 0.027914809 | 0.551250982 |
|  | COX2 (PTGS2) | -0.038813629 | | 0.407279665 | -0.231335274 | 5.58E-07 |
| M2 Macrophage | CD163 | -0.030173811 | | 0.519491262 | -0.288795066 | 3.01E-10 |
|  | VSIG4 | -0.015500132 | | 0.740772364 | -0.237076855 | 2.85E-07 |
|  | MS4A4A | -0.031568033 | | 0.500370304 | -0.298778428 | 6.74E-11 |
| Neutrophils | CD66b | 0.196637907 | | 2.25E-05 | 0.18531645 | 6.62E-05 |
|  | CD11b (ITGAM) | -0.014478261 | | 0.757307996 | -0.248390124 | 7.21E-08 |
|  | CCR7 | 0.098321432 | | 0.035420378 | -0.179738552 | 0.00010991 |
| Natural killer cell | KIR2DL1 | -0.072238207 | | 0.122646993 | -0.255819819 | 2.82E-08 |
|  | KIR2DL3 | -0.085018921 | | 0.069093988 | -0.237208569 | 2.81E-07 |
|  | KIR2DL4 | -0.15657415 | | 0.000772576 | -0.369695423 | **2.79E-16** |
|  | KIR3DL1 | -0.129295636 | | 0.005586271 | -0.276911236 | 1.66E-09 |
|  | KIR3DL2 | -0.122960744 | | 0.008431416 | -0.242090328 | 1.56E-07 |
|  | KIR3DL3 | -0.083185072 | | 0.07532885 | -0.130255863 | 0.005240229 |
|  | KIR2DS4 | -0.060074971 | | 0.199384717 | -0.18769214 | 5.30E-05 |
| Dendritic cell | HLA-DPB1 | | -0.052607393 | 0.261204439 | -0.319306161 | **2.58E-12** |
|  | HLA-DQB1 | | -0.104254931 | 0.025671926 | -0.212795023 | 4.34E-06 |
|  | HLA-DRA | | -0.085476904 | 0.067604678 | -0.388714262 | **5.71E-18** |
|  | HLA-DPA1 | | -0.046005013 | 0.32591267 | -0.339065694 | **8.75E-14** |
|  | BDCA-1  (CD1C) | | 0.164734389 | 0.000400051 | 0.044470402 | 0.371464613 |
|  | BDCA-4 (NRP1) | | -0.013532083 | 0.772718306 | -0.257148071 | 1.49E-07 |
|  | CD11c (ITGAX) | | 0.02026287 | 0.665375331 | -0.23118486 | 2.50E-06 |
| **Th1** | STAT4 | | -0.024751934 | 0.597257866 | -0.206574049 | 2.73E-05 |
|  | STAT1 | | -0.073458082 | 0.116439037 | -0.336801607 | **3.17E-12** |
|  | IFN-γ (IFNG) | | -0.1031968 | 0.027218366 | -0.31505943 | **8.31E-11** |
|  | TNF-α (TNF) | | 0.056982923 | 0.22354925 | -0.103011114 | 0.038010722 |
| **Th2** | GATA3 | | 0.045927882 | 0.326725294 | -0.10113681 | 0.04167086 |
|  | STAT6 | | -0.009939233 | 0.83200339 | 0.028339028 | 0.56910547 |
|  | STAT5A | | -0.023233055 | 0.619952756 | 0.045165836 | 0.364024181 |
|  | IL13 | | 0.084416182 | 0.07109486 | -0.058887876 | 0.236441924 |
| **Tfh** | BCL6 | | -0.105366236 | 0.024129917 | -0.242976021 | 7.23E-07 |
|  | IL21 | | -0.010646026 | 0.820253653 | -0.132185956 | 0.007653767 |
| **Th17** | STAT3 | | 0.054689671 | 0.242772053 | -0.100447613 | 0.043089471 |
|  | IL17A | | 0.17939451 | 0.000113351 | 0.077288206 | 0.119984372 |
| **Treg** | FOXP3 | | 0.125914209 | 0.006974406 | -0.125008753 | 0.011702626 |
|  | CCR8 | | 0.115122017 | 0.013694165 | -0.150115802 | 0.00242436 |
|  | STAT5B | | 0.308003196 | **2.27E-10** | 0.240996758 | 8.94E-07 |
|  | TGF β(TGFB1) | | 0.053670644 | 0.280642561 | -0.252316547 | 2.58E-07 |
| **T cell exhaustion** | PD-1 (PDCD1) | | -0.06349795 | 0.201675563 | -0.258552085 | 1.27E-07 |
|  | CTLA4 | | 0.033689071 | 0.498459747 | -0.255107244 | 1.88E-07 |
|  | LAG3 | | -0.10610705 | 0.032561947 | -0.329053588 | **1.05E-11** |
|  | TIM-3 (HAVCR2) | | -0.02547340 | 0.608808162 | -0.330337224 | **8.60E-12** |
|  | GZMB | | 0.334110968 | **4.81E-12** | 0.069813942 | 0.160292506 |

**Table S12. Correlation analysis between UMODL1 and biomarkers in COAD.**

| **Description** | **Gene markers** | **COAD** | |
| --- | --- | --- | --- |
|  |  | **Cor** | **Pvalue** |
| **Natural killer cell** | KIR2DL4 | -0.35 | 1.9e-09 |
| **Dendritic cell** | HLA-DPB1 | -0.32 | 8.1e-08 |
|  | HLA-DRA | -0.33 | 2.6e-08 |
| **T cell exhaustion** | TIM-3 (HAVCR2) | -0.30 | 4.3e-07 |
|  | LAG3 | -0.33 | 2.1e-08 |

**Table S13. Correlation analysis between OIT3 and biomarkers in COAD.**

| **Description** | **Gene markers** | **COAD** | |
| --- | --- | --- | --- |
|  |  | **Cor** | **Pvalue** |
| **Treg** | STAT5B | 0.37 | 1.5e-10 |
| **T cell exhaustion** | GZMB | 0.37 | 2.9e-10 |
